# Supplementary material for: The presence of knockdown resistance mutations reduces male mating competitiveness in the major arbovirus vector, Aedes aegypti
Source: PLoS Negl Trop Dis. 2021 Feb 5;15(2):e0009121. doi: 10.1371/journal.pntd.0009121 (PMC7891746; doi:10.1371/journal.pntd.0009121)
Supplement: S1 Table — (DOCX) [file pntd.0009121.s001.docx]

| **Assay No.** | **Male # 1** | **Male # 2** | **Female** | **Replicates** |
| --- | --- | --- | --- | --- |
| 1 | Rho B S-Cairns | R-BC | R-BC | 3 |
| 2 | Rho B S-Cairns | R-BC | S-Cairns | 3 |
| 3 | Rho B S-Cairns | R-TL | R-TL | 3 |
| 4 | Rho B S-Cairns | R-TL | S-Cairns | 3 |
| 5 | Rho B R-TL | S-Cairns | R-TL | 3 |
| 6 | Rho B R-TL | S-Cairns | S-Cairns | 3 |
| 7 | Rho B R-TL | R-BC | R-TL | 3 |
| 8 | Rho B R-TL | R-BC | R-BC | 3 |
| 9 | Rho B R-BC | R-TL | R-TL | 3 |
| 10 | Rho B R-BC | R-TL | R-BC | 3 |
| 11 | Rho B R-BC | S-Cairns | R-BC | 3 |
| 12 | Rho B R-BC | S-Cairns | S-Cairns | 3 |
