# Supplementary material for: The presence of knockdown resistance mutations reduces male mating competitiveness in the major arbovirus vector, Aedes aegypti
Source: PLoS Negl Trop Dis. 2021 Feb 5;15(2):e0009121. doi: 10.1371/journal.pntd.0009121 (PMC7891746; doi:10.1371/journal.pntd.0009121)
Supplement: S2 Table — (DOCX) [file pntd.0009121.s002.docx]

| **Strain** | **N tested** | **Fed** | **Unfed** | **% Fed** |
| --- | --- | --- | --- | --- |
| S-Cairns | 30 | 30 | 0 | 100% |
| R-TL | 30 | 30 | 0 | 100% |
| R-BC | 30 | 29 | 1 | 96.7% |
