# Supplementary material for: The presence of knockdown resistance mutations reduces male mating competitiveness in the major arbovirus vector, Aedes aegypti
Source: PLoS Negl Trop Dis. 2021 Feb 5;15(2):e0009121. doi: 10.1371/journal.pntd.0009121 (PMC7891746; doi:10.1371/journal.pntd.0009121)
Supplement: Attachment — Submitted filename: Revisions Letter-Rigby et al-PLOS NTD.docx [file pntd.0009121.s004.docx]

|  | **JOINT CAPABILITIES GROUP**  JOINT HEALTH COMMAND  AUSTRALIAN DEFENCE FORCE MALARIA AND INFECTIOUS DISEASE INSTITUTE  Weary Dunlop Drive, Gallipoli Barracks, ENOGGERA QLD 4051 |
| --- | --- |

Dear Felix Hol and Elvina Viennet,

We would like to thank you and the reviewers for a thorough and positive assessment of our manuscript. We have made the corrections and additions to the main text and figures to address the comments as thoroughly as possible. Please find below the original comments in italic font and our responses in regular font. We have also highlighted the changes in the main text in yellow.

*Reviewer #1: This study was done well and the manuscript was well written. It provides specific evidence enlightening the reader about a cost of insecticide resistance that was missing from the literature. The main questions that arose while I was reading the manuscript (e.g. changes in body size) were addressed in the discussion, which indicates the authors were thorough when considering the strengths, weaknesses, and implications of their work. I have only a few suggestions, and they are all very minor.*

*Line 128-129: Can the authors please provide the manufacturer’s information for this artificial membrane feeder system or describe how it is made.*

We have now referenced the manuscript: 26. Finlayson C, Saingamsook J, Somboon P. A simple and affordable membrane-feeding method for *Aedes aegypti* and *Anopheles minimus* (Diptera: Culicidae). Acta Trop. 2015;152: 245–251. doi:10.1016/j.actatropica.2015.09.026.

This publication reports on the use of a simple membrane feeder system in which a petri dish containing blood is covered with Parafilm M, stretched thinly over the top of the dish. This system of artificial blood feeding was utilised in the current manuscript. We now state (L129) “They were blood-fed once per week using an artificial membrane feeding system [26] and defibrinated sheep blood (Serum Australis)”.

*Line 188: The interplay between numeric characters in this sentence seems awkward. Consider rephrasing here and in other places to “Pools of 10 female mosquitoes, 3-4 days old, from….”*

We have now re-worded the sentence which reads (L189) “Pools of 10 female mosquitoes, 3-4 days old, from the S-Cairns, R-TL and R-BC strains were deprived of sugar solution for 24 h before testing for avidity.”

*Line 190-191: Can the authors please clarify whether the same volunteer was used each time or whether different volunteers were used.*

We have clarified that only one human volunteer was used for the blood-feeding behaviour experiments in the text which now reads, (L194) “Three batches of 10 mosquitoes from each strain were used for blood-feeding experiments and offered blood from the arm of the same human volunteer.”

*Line 317-325: Can the authors please add a brief sentence or two to this discussion regarding whether the change in WBFs observed between their strains match what would be expected based on the differences in body size found in other studies.*

Although a significant decrease in WBF was noted with smaller R-BC males, there was no general relationship between smaller body size associated with insecticide-resistant *Ae. aegypti* and WBF. This is consistent with previous studies that report no impact of size on WBF in this species (Mukundarajan et al. 2017, Villarreal et al. 2017). We added the following sentences to the manuscript (L320) “In *Ae. aegypti*, there are conflicting studies on the relationship between body size and WBF, reporting either no relationship [36,37] or that smaller mosquitoes have lower WBF frequencies [28].” (L327) “Although a significant decrease in WBF was noted with smaller R-BC males, there was no general relationship between the smaller body size associated with insecticide-resistant *Ae. aegypti* [14,21] and WBF.”

*Line 364-365: Please add the date this information was cited as per the reference guide for this journal*

Done. (L370) “2. WHO. Dengue and severe dengue. 2020 [cited 5 Aug 2020]. Available: https://www.who.int/news-room/fact-sheets/detail/dengue-and-severe-dengue.

*In there references there is an inconsistent use of upper-case letters in journal titles. Please tidy these up a bit. I believe the journal guidelines ask for sentence case usage.*

Done.
 *I have a slight concern about the use of red and green in the same figures as readers who are red-green colourblind may have difficulty with these. Please consider changing one of these colours*.

We acknowledge the concerns presented by the reviewer in reference to Fig 2 and 3 which both use the colours red and green on the same graph. We believe that we have already addressed the potential difficulty for some readers to distinguish between these two colours by, labelling the columns with text in Fig 2 and the utilisation of different symbols in addition to colours used in Fig 3.

*Reviewer #2: Rigby and colleagues have demonstrated a cost of insecticide resistance in Aedes aegypti associated with an isolated kdr genotype, which manifests as a reduction in male mating success. As Aedes control relies heavily on the use of insecticides but the rapid evolution of resistance to these chemicals compromises their efficacy, the conservation or restoration of insecticide susceptibility in Ae. aegypti populations is of great importance. The manuscripts is well written, and the figures and tables are very clear. Great job.*

*One thing I was wondering while reading the manuscript was whether females mate with multiple males. If so, how would that have affect the outcome?*

The mating competition method and protocol used in this study assumes that female mosquitoes become refractory to a second mating and only mate once. The authors acknowledge that studies exist demonstrating that re-insemination of female *Ae. aegypti* may occur in some situations. Given that multiple insemination events for a single female are possible, however are reported as a rare event, the authors of this manuscript utilised a robust mating competition protocol and reciprocal assays (swapping the Rho B stained strains) to ensure that there was no labelling bias. If females were frequently inseminated more than once, with both unstained and stained males, we would predict that Rho B staining would be present in the majority of dissected females. This was not the case as demonstrated in the raw data included as supplementary material, Data S1.

*Minor comments:*

*- Omit the p-value from the abstract*

We have now removed the p-value from the abstract as requested.

*- Provide details on the ‘artificial membrane feeding system’*

As above, this publication reports on the use of a simple membrane feeder system in which a petri dish containing blood is covered with Parafilm M, stretched thinly over the top of the dish. The reported system of blood feeding reported above was ultilised in this study. We now state (L129) “They were blood-fed once per week using an artificial membrane feeding system [26] and defibrinated sheep blood (Serum Australis)”

*- “ (…) effects that have been previously described, may encourage reversion to susceptibility in the absence of insecticide selection pressures”. Yes, but only if the fitness costs are detrimental.*

The authors acknowledge this statement. To clarify the authors intent, we remove the word “effects” and replace with “fitness costs” in this sentence to ensure that the message reflects that the statement only holds true for effects of resistance that are detrimental to the mosquito population. The sentence now reads (L50) “These consequences of resistance evolution, especially when combined with other pleiotropic fitness costs that have been previously described, may encourage reversion to susceptibility in the absence of insecticide selection pressures.”

*- Host-location experiment: I assume this falls under the same ethics approval (P2273)*

Yes. We have clarified this in the text which now reads (L201) “Mosquitoes were allowed to acclimatise and rest for 10 min before a human observer entered and sat in the middle of the room (QIMR Berghofer Human Research Ethics approval P2273).”

*Reviewer #3: The submitted manuscript by Rigby et al reports a reduction in mating success in Ae. aegypti males (R-BC strain) associated with two mutations in the voltage gated sodium channel that confer insecticide resistance. Males of the R-BC strain, that was generated by backcrossing an insecticide resistant strain carrying the two VGSC mutations to an insecticide susceptible strain, also showed a lower wing beat frequency that authors hypothesize could be related to the reduced mating success. However, the underlying causes for the reduction in mating success seem to be more complicated, given also the observation that the parental insecticide resistant strain has a much higher wing beat frequency than the R-BC males, but also a reduced mating success phenotype. Maybe the authors could comment on this a bit more. Overall I found the manuscript well written and it does present some interesting aspects of the potential fitness costs of VGSC mutations.*

While we acknowledge that we could draw further conclusions of the relationship between the increased wing beat frequency recorded in the parental insecticide-resistant strain that also recorded a reduced mating success phenotype, we refrain from doing so in this manuscript. The authors acknowledge in the manuscript text that the underlying causes of mating success require further investigation along with its relationship with wing beat frequency. This presents an interesting and intriguing opportunity for further research in the future, however, is beyond the scope of the current study.

*I have some minor points below.*

*Mating success experiment: please add the following information: what was the efficiency of Rho B labelling in males*

We have now provided clarification in the text that all males were able to be visually confirmed for the presence of Rho B labelling at the conclusion of the 96 h labelling period. Those male mosquitoes that failed to take a Rho B/Sucrose meal during this period most likely died before the conclusion of the labelling period. Male mosquitoes were not offered a sucrose meal post-emergance prior to Rho B labelling. The text in the manuscript now reads (L144) “At the conclusion of the 96 h labelling period, 100% of the Rho B fed males were confirmed for labelling success by visual inspection.”

*and what was the percentage of females that had not mated.*

*The raw data used to generate the graphs are not provided. I think they should be part of the supplementary information.*

We now include all raw data used for the mating competition experiments as supporting information (Data S1). This information includes a record of the rare occasions where females that had not be inseminated were recorded. Text within the manuscript now reads (L217) “The numerical data used in all figures are included in Data S1.”

*Lines 311-313: a reference is needed*

Done. The reference Cator et al., 2009 (24) was added as requested. (L315)

*Line 314: check the sentence*

Completed. The sentence now reads (L316) “Therefore, our observations of a reduced WBF of males from the resistant backcrossed R-BC strain, in comparison to the susceptible parental strain, indicate that pleiotropic effects of kdr on male WBF may have contributed to the reduced mating success of this strain.”

*Figure Files:
While revising your submission, please upload your figure files to the Preflight Analysis and Conversion Engine (PACE) digital diagnostic tool, https://pacev2.apexcovantage.com. PACE helps ensure that figures meet PLOS requirements. To use PACE, you must first register as a user. Then, login and navigate to the UPLOAD tab, where you will find detailed instructions on how to use the tool. If you encounter any issues or have any questions when using PACE, please email us at* [*figures@plos.org*](mailto:figures@plos.org)*.*

Acknowledged

*Data Requirements:
Please note that, as a condition of publication, PLOS' data policy requires that you make available all data used to draw the conclusions outlined in your manuscript. Data must be deposited in an appropriate repository, included within the body of the manuscript, or uploaded as supporting information. This includes all numerical values that were used to generate graphs, histograms etc.. For an example see here:* [*http://www.plosbiology.org/article/info%3Adoi%2F10.1371%2Fjournal.pbio.1001908#s5*](http://www.plosbiology.org/article/info%3Adoi%2F10.1371%2Fjournal.pbio.1001908#s5)*.*

We now include all numerical values that were used to generate graphs as supporting information. Text within the manuscript now reads (L217) “The numerical data used in all figures are included in Data S1.” *Reproducibility:
To enhance the reproducibility of your results, PLOS recommends that you deposit laboratory protocols in protocols.io, where a protocol can be assigned its own identifier (DOI) such that it can be cited independently in the future. For instructions see* [*http://journals.plos.org/plosntds/s/submission-guidelines#loc-materials-and-methods*](http://journals.plos.org/plosntds/s/submission-guidelines#loc-materials-and-methods)

Acknowledged

End of comments and responses.

Thank you for your consideration.

Kind regards,

**LM Rigby** (on behalf of all co-authors)

Vector Surveillance and Control

Australian Defence Force Malaria and Infectious Disease Institute

Building K10, Weary Dunlop Drive, Gallipoli Barracks, Enoggera, 4051, QLD, Australia

email: lisa.rigby@defence.gov.au, lisa.rigby@uqconnect.edu.au

phone: 07 3332 2358

13 December 2020
